# Epidemiology, histopathology, clinical outcomes and survival of 50 cases of appendiceal mucinous neoplasms: Retrospective cross-sectional single academic tertiary care hospital experience

**DOI:** 10.1016/j.amsu.2021.102199

**Published:** 2021-03-06

**Authors:** Ammar Aleter, Walid El Ansari, Ali Toffaha, Adham Ammar, Fakhar Shahid, Abdelrahman Abdelaal

**Affiliations:** a Department of General Surgery, Hamad General Hospital, Hamad Medical Corporation, Doha, Qatar; b Department of Surgery, Hamad General Hospital, Hamad Medical Corporation, Doha, Qatar; c College of Medicine, Qatar University, Doha, Qatar; d School of Health and Education, University of Skovde, Skovde, Sweden; e Department of Laboratory Medicine and Pathology, Hamad General Hospital, Doha, Qatar

**Keywords:** Appendix, Mucinous neoplasm, Mucinous adenocarcinoma, Pseudomyxoma peritonei, Low-grade appendiceal mucinous neoplasm, Mucocele of appendix

## Abstract

**Background:**

Appendicular neoplasms are rare, most commonly as carcinoids followed by appendicular mucinous neoplasms (AMN). To date, there remains controversy regarding the best treatment of AMN and factors affecting its prognosis.

**Method:**

Retrospective chart review of patients operated for appendicular pathology (January 2011–December 2018, follow up to December 2020) at our institution. For all AMN patients, data included pre-operative clinical presentation, and operative/post-operative findings.

**Results:**

12454 patients underwent appendectomy, of whom 50 (0.4%) had AMN histopathologically (mean age = 47.2). Most patients had laparoscopic appendectomy as primary surgery. Low grade AMN was the most common subtype (n = 41, 82%), and pseudomyxoma peritonei (PMP) was found in 8 (16%) patients. Based on histopathology and margin involvement, the 50 patients were categorized into 3 prognostic categories of recurrence risk (no risk, 24 patients; low risk, 8; high recurrence risk, 18 patients). Disease-free survival (DFS) was lowest for high recurrence risk group (P < 0.001). Eleven (22%) patients had AMN involving resection margin, of whom 3 had no completion surgery and had no recurrence. Higher tumor markers were associated with lower DFS, however it was not statistically significant.

**Conclusion:**

AMNs are rare but serious due to the risk of PMP. Laparoscopic approach for AMN may be feasible. Prognostic categories were significantly inversely correlated with recurrence risk; hence useful in predicting prognosis. Contrary to previous proposals, AMNs with acellular mucin at margin or local acellular mucin spillage may not require secondary surgery, especially if the patient is in low recurrence risk group. Tumor markers may predict risk of recurrence.

## Highlights

• Laparoscopic approach may be feasible for appendiceal mucinous neoplasms (AMN).
• AMN can be categorized into 3 risk recurrence groups (No risk, low, and high).
• AMN with acellular mucin at the margin may not require secondary surgery.
• Higher tumor markers may indicate high risk of AMN recurrence.

## 1 Introduction

Mucinous tumors may originate from several sites including appendix, ovary, colon, pancreas and gallbladder [1]. Despite that appendiceal tumors are rare (about 1% of all appendectomies), appendiceal mucinous neoplasms (AMNs) are the second most common tumors that affect the appendix after carcinoid tumors [2].

Appendiceal mucocele (AM) is a morphological description of the distended, mucus-filled appendix [3]. AM is an ambiguous term that usually describes an imaging finding rather than a pathologic diagnosis [3]. AM has widely variable behavior, ranging from non-neoplastic to neoplastic [3]. Such uncertain malignant potential and the possibility of recurrence has led to many histologic classifications.

AM lesions are generally divided into two broad categories [4]. The first, non-neoplastic appendiceal mucinous lesions (simple mucocele), are characterized by degenerative epithelial changes and distention, with no evidence of mucosal hyperplasia or neoplasia [4]. The second, neoplastic appendiceal mucinous lesions, is further subdivided into serrated polyps of the appendix and AMN. AMNs are sub-classified into (LAMNs), high-grade appendiceal mucinous neoplasms (HAMNs) and mucinous adenocarcinomas [4]. The most prevalent subtype of AMNs is LAMN [5].

AMNs are enigmatic tumors of unpredictable recurrence [6], although the recurrence rate of LAMN is very low if removed intact. Conversely, patients with positive margin, appendiceal rupture, mucin or cells outside the appendix have significantly higher possibility of developing malignant pseudomyxoma peritonei (PMP) [[7], [8], [9], [10]], where there is malignant spread with high relapse of disseminated intraperitoneal mucinous tumors and free mucin [1,11,12].

The literature reveals knowledge gaps. There remains controversy regarding the surgical treatment of AMN, particularly the ideal management of a positive margin. Appendectomy alone is sufficient if the tumor is confined to the appendix [3]. However, if the peri-appendiceal margin is involved by neoplastic epithelium or acellular mucin, some authors suggest more extensive resection (right-sided hemicolectomy or caecectomy) [13], while others found that such margin involvement does not predict the recurrence and recommend a conservative approach [10]. Very few papers have been published from the Middle East and North Africa (MENA) region describing AMN, its full picture and possible prognostic factors (including histologic staging) and the associations of such variables with the tumor's biological behavior [14,15].

Therefore, the current study used the most recent and most widely accepted classification of AM described by Peritoneal Surface Oncology Group International (PSOGI) [4]. We assessed the prevalence, clinical presentation, diagnostic imaging, treatment and survival of AM, particularly AMN. The specific objectives were to assess AMN's:

- Range of demographic, clinical, histopathological and surgical characteristics
- Cases with controversial management guidelines, their treatment and outcome
- Distribution of patients over three prognostic groups and the disease-free survival (DFS) of each group
- The association between tumor marker levels and DFS.

## 2 Methods

Ethical approval was obtained from the Institutional Review Board, Medical Research Center (IRB#17167/17) at Hamad Medical Corporation (HMC), Qatar. The current retrospective chart review is of all patients at HMC with suspected clinical and/or radiological appendicular pathology who underwent elective or emergent surgery with intention to treat between January 2011 to December 2018 with a follow up to December 2020. A total of 12454 patients were eligible, and their data searched for histopathological diagnosis of AMN in order to determine the prevalence. Of the 12454, only 50 patients found to have histopathologically confirmed AMN. These 50 cases underwent a comprehensive clinico-pathological analysis for demographics, clinical presentation, modality of diagnosis, investigations (ultrasonography (US), CT scan and histological findings), tumor type and size, margins and lymph node involvement, tumor staging and differentiation (eighth edition American Joint Committee on Cancer (AJCC) staging criteria] [16], operative/post-operative findings, and type of treatment and follow up. We report this study in line with STROCSS criteria (strengthening the reporting of cohort studies in surgery) [17].

## 3 Statistical analysis

Descriptive statistics in the form of mean and standard deviation for continuous variables such as age in years and mucocele length and width in cm and frequency with percentage for categorical variables were performed. Kaplan Meier DFS curves were presented for follow up data from January 2011 to December 2020. Patients data were right censored. Kaplan Meier for overall DFS (months) was presented and DFS stratified into 3 prognostic categories of recurrence risk: curative (n = 24), low recurrence risk (n = 8), and high recurrence risk (n = 18), DFS of patients with normal and abnormal CEA, and DFS of patients with normal and abnormal CA 19-9 were also performed. To see significant difference in DFS among categories log-rank test was applied. P value 0.05(two tailed) was considered statistically significant difference. Data analysis was carried out using the Statistical Package for Social Sciences version 20 (SPSS Inc., Chicago, IL, USA).

## 4 Results

Table 1 shows the patients' demographic and clinical characteristics. The mean age was 47.2 (range 19–77 years), with near equal proportions of males and females. The predominant (52%) nationalities were of Middle Eastern descendent, and most (70%) patients did not have comorbidities. The main presenting symptom was localized pain (83.3%) followed by vomiting (31%), and only 7% had fever. The majority of patients had abdominal distention, about three quarters (72.5%) had localized tenderness, but a palpable mass was felt in less than one fifth (18%) of the patients.

**Table 1:** Demographic and clinical characteristics of the sample.

Variable	N (%)
**Demographic**	
Age (years, mean ± SD)	47.2 ± 13.1
Sex (n = 50)	
Female	23(46)
Male	27(54)
Nationality (n = 50)	
Southeast Asian^a^	6(12)
Middle East	26(52)
South Asian^b^	12(24)
African	2(4)
European	4(8)
Comorbidities (n = 50)	
No	35(70)
Yes^c^	15(30)
**Symptoms**	
Pain (n = 47)	
None^d^	4(8.3)
Localized	40(85)
Generalized	3(6.4)
Fever (n = 43)	
No	40(93.02)
Yes	3(6.97)
Nausea/vomiting (n = 42)	
No	29(69)
Yes	13(31)
Loss of weight (n = 39)	
No	37(94.87)
Yes	2(5.1)
Anorexia (n = 29)	
No	22(84.6)
Yes	4(15.4)
Diarrhea (n = 22)	
No	20(90.9)
Yes	2(9.1)
Vaginal bleeding (n = 50)	
No	49(98)
Yes^e^	1(2)
**Examination**	
Tenderness (n = 40)	
No	7(17.5)
Localized	29(72.5)
Generalized	4(10)
Palpable mass (n = 39)	
No	32(82.0)
Yes	7(17.9)
Abdominal distention (n = 36)	
No	32(88.8)
Yes	4(11.1)
Recurrent cutaneous fistula (n = 50)	
No	49(98)
Yes^f^	1(2)

a All were Philippines.

b India, Pakistan, Nepal, Bangladesh.

c Includes diabetes mellitus, hypertension, asthma, treated primary colon cancer, osteoarthritis, end stage renal disease, dyslipidemia.

d No presenting symptoms, discovered incidentally during inguinal hernia (1), colonoscopy (1), during TAH + BSO for initial diagnosis by US abdomen as ovarian cystic neoplasm, TAH + BSO was aborted and appendectomy done instead (1), routine physical examination (1).

e Patient presented with vaginal bleeding and initial diagnosis by MRI was ovarian cystic neoplasm, appendectomy done with TAH + BSO for the adherent and enlarged appendix and the origin of the mass turned out to be AMN.

f Patient treated initially as appendicular mass, treated conservatively with follow up planned interval appendectomy. At surgery, extensive adhesions and abscess collections were found and appendix could not be identified. Enterocutaneous fistula developed post operatively and patient was diagnosed as mucinous adenocarcinoma after colonoscopy. Patient received cytoreductive surgery with hyperthermic intraperitoneal chemotherapy (CRS + HIPEC); TAH: total abdominal hysterectomy; BSO: bilateral salpingo-oophorectomy.

Table 2 illustrates that less than half the sample had leukocytosis, while more than a third (39%) were anemic. CT abdomen was the most used imaging method for diagnosis (79.54%). US abdomen could identify a suspicious heterogenous mass in 5 out of 14 patients (35.7%), but further imaging (CT scan or MRI) was needed to characterize such heterogenous masses. The appendix could not be visualized in 4 patients who underwent US abdomen.

**Table 2:** Laboratory and imaging characteristics of the sample.

Variable	N (%)
**Leukocytosis**^a^ (n = 39)	
No	20(51.3)
Yes	19(48.7)
**Anemia**^b^ (n = 39)	
No	28(71.8)
Yes	11(38.2)
**US abdomen** (n = 48)	
Not visualized^c^	4(8.33)
Appendicitis	2(4.16)
Appendicitis with collection	3(6.25)
Heterogenous mass	5(10.41)
Not done	34(70.83)
**CT abdomen** (n = 44)	
Appendicitis	4(9.09)
Appendicitis + collection or free fluid	11(25)
Mucocele	19(43.18)
Mesenteric cyst	1(2.27)
Not done	9(20.45)

a >11000/mm^3^.

b Hb < 13.5 men, <12 women.

c Identification of appendix was not possible; US: ultrasound; CT: computerized tomography.

Table 3 depicts the type of surgeries, intraoperative findings and histological characteristics. Most primary surgeries comprised appendectomy, with open to laparoscopic ratio of around 1:2.5. In only 3 (6%) patients, laparoscopic had to be converted to open, and laparoscopic partial cecectomy was performed in one patient due to involvement of the cecum by the mucocele. In one patient, the mucocele of the appendix was found incidentally during repair of indirect inguinal hernia (Amyand hernia), and in another, appendectomy was aborted due to finding of a huge adherent mass along with multiple peritoneal seeds. Right hemicolectomy was the primary surgery for 4 patients because of cecal involvement or large adherent mass. The majority of patients (67.7%) had intact mucocele and 8.8% of the patients had metastasis at primary surgery.

**Table 3:** Surgical and histological characteristics of the sample.

Variable	N (%)
**Primary surgery (n = 50)**	
**Type**	
Open appendectomy	11(22)
Laparoscopic appendectomy	28(56)
Laparoscopic converted to open appendectomy	3(6)
Laparoscopic appendectomy + partial cecectomy	1(2)
Right Hemicolectomy	4(8)
Appendectomy during inguinal hernia repair	1(2)
Diagnostic laparoscopy^a^	1(2)
Laparotomy, TAH + BSO + appendectomy	1(2)
**Findings** (n = 35)	
Intact mucocele^b^	24(67.65)
Mucocele spillage^c^	8(23.5)
Peritoneal seeding^d^	3(8.8)
**Margins post primary surgery** (n = 50)	
Free margins	37(74.0)
Margins involved	11(22.0)
Dysplasia at resection margin^e^	1(2.0)
Peritoneal biopsy showing malignant nodule^f^	1(2.0)
**Submitted Lymph nodes** (n = 50)	
Specimens contain lymph nodes^g^	6(12.0)
Specimens do not contain lymph nodes	44(88.0)
**Submitted mucocele** (n = 50)	
**Length** cm Mean ± SD50	5.9 ± 4.5
<5 cm	9(18.0)
≥5 - <10	13(26.0)
≥10 - <15	4(8.0)
≥15 - <20^h^	2(4.0)
Could not be assessed	22(44.0)
**Width** cm (n = 50)	
Mean ± SD	4.25 ± 4.2
<5 cm	14(28.0)
≥5 - <15	2(4.0)
≥15	1(2.0)
Could not be assessed	33(66.0)
**Histological type (AJCC 8th edition)** (n = 50)	
Low-grade appendiceal mucinous neoplasm	41(82.0)
Mucinous adenocarcinoma	5(10.0)
Appendiceal adenoma	3(6.0)
Neuroendocrine tumor in background of mucocele^i^	1(2.0)
**PMP discovered during primary surgery or follow up** (n = 50)	
Not present	42(84.0)
Present	8(16.0)
**Secondary surgery** (n = 16)	16(32.0)
CRS + HIPEC	9(56.25)
Laparoscopic partial stapled cecectomy^j^	1(6.25)
Laparoscopic right hemicolectomy^k^	2(12.5)
Oncological right hemicolectomy	3(18.75)
Laparoscopic exploration + excision of appendicular stump^l^	1(6.25)

a Appendectomy not done, only diagnostic biopsy from peritoneal seeding followed by cytoreductive surgery + HIPEC as second surgery.

b No spillage found.

c Perforated, ruptured appendix or localized gelatin collection.

d Peritoneal seeding present either with spillage or intact mucocele.

e Histopathology of primary surgery showed appendiceal adenoma, margins not involved but dysplasia at the resection margin found (completion laparoscopic partial cecectomy done).

f One sample was from peritoneal biopsy showing malignant seeding.

g All reactive lymph nodes, no malignant invasion.

h One was 15 cm and the other was 17 cm.

i Specimen contained neuroendocrine tumor (carcinoid) combined with acellular mucin within the muscularis propria, epithelial atypia and denuded epithelial lining concerning for mucinous cystadenoma, LAMN ruled out due to absence of dysplasia.

j Second surgery done for Low grade dysplasia found at the resection margin.

k Second surgery done for: LAMN with T4a TNM staging, LAMN with involved resection.

l 1 year after primary open appendectomy patient developed stump appendicitis with gelatinous collection found during exploration; TAH: total abdominal hysterectomy; BSO: bilateral salpingo-oophorectomy; CRS: cytoreductive surgery; HIPEC: hyperthermic intraperitoneal chemotherapy.

Margins were free post primary surgery in almost three quarters of the patients, whilst it was involved in 11 patients (23.4%) and all of whom had appendectomies. The decision for nine patients with margins involvement was to go for secondary surgery while the other 2 were followed closely. The average length and width of the excised mucocele was 5.9 ± 4.5 and 4.25 ± 4.2 cm respectively.

LAMN was the most common histological type (82%), and one mucocele composed of a rare type of neuroendocrine tumor (carcinoid) associated with acellular mucin reaching the muscularis propria, hence mucinous cystadenoma could not be ruled out as a second combined tumor. Five patients (10%) were found to have mucinous adenocarcinoma and all of them had peritoneal metastasis on presentation or developed PMP, hence they referred for cytoreductive surgery with hyperthermic intraperitoneal chemotherapy (CRS + HIPEC).

Pseudomyxoma peritonei was present during primary surgery or follow up for 8 patients (16%). Secondary surgery was done for total of 16 patients (32%), 9 of them (56.3%) had CRS + HIPEC and another 7 (43.7%) underwent other types of secondary surgery which was mainly for margins involvement or high-grade tumors.

Table 4 depicts that 40% of patients were stage Tis, and another 20% had the tumor confined to the appendix (T1, T2, T3). Eight patients staged as T4, and 4 of those were referred for CRS + HIPEC because of associated PMP. Another two patients were treated with right hemicolectomy, and the remaining two patients were pT4a and were followed for 2 and 4 years respectively with no recurrence. Carcinoembryonic antigen (CEA) was elevated in 8 patients (16%), and cancer antigen (CA)19-9 was elevated in 14% of patients. Alpha-fetoprotein (AFP) was normal across the sample. A total of 35 patients (70%) were followed for at least 1 year post primary surgery and only 15 patients (30%) were lost follow up within <1 year of surgery.

**Table 4:** Staging, tumor marker and survival characteristics of the sample.

Variable*	N (%)
**TNM stage**	
T	
T1	2(4)
T2	4(8)
T3	4(8)
T4	8(16)
Tis	20(40)
Tx	9(18)
No invasion (adenoma)	3(6)
N	
N0	20(40)
N1	0(0)
Nx	30(60)
M	
M0	23(46)
M1	12(20)
Mx	15(30)
**Tumor markers**	
CEA^a^	
Normal	27(54.0)
Elevated	8(16.0)
Not Done	15(30.0)
CA 19-9^b^	
Normal	24(48.0)
Elevated	7(14.0)
Not Done	19(38.0)
CA 125^c^	
Normal	8(16.0)
Elevated	1(2.0)
Not Done	41(82.0)
AFP^d^	
Normal	14(28.0)
Not Done	36(72.0)
**Survival (years)**	
Overall	
0	15(30.0)
1	9(18.0)
2	6(12.0)
3	3(6.0)
4	8(16.0)
5	3(6.0)
6	4(8.0)
7	2(4.0)
Disease free	
0	18(36.0)
1	11(22.0)
2	5(10.0)
3	3(6.0)
4	7(14.0)
5	2(4.0)
6	2(4.0)
7	2(4.0)

* All variables based on data from 50 cases.

a Normal reference value < 5 ng/ml.

b Normal reference value < 27 U/mL.

c Normal reference value < 35 U/mL.

d Normal reference value < 10 ng/ml.

Table 5 displays the prognostic distribution of the sample. The prognostic classification comprised three (curative, and low/high risk of recurrence) groups according to AJCC 8th edition and PSOGI [4,16]; additional treatment that was provided (where indicated); and follow up. The curative group included almost half (24) the sample according to their histopathology, no additional surgery was undertaken (apart from primary appendectomy) and follow up did not show recurrence. The low risk of recurrence group had 8 patients (6 had secondary surgery, 2 had their LAMN resected intact but the margins were positive for acellular mucin), and follow up showed no recurrence. The high-risk of recurrence group had 18 patients, one patient had spillage of acellular mucin and was closely followed for >1 year with no recurrence.

**Table 5:** Clinical characteristics of the three prognostic categories of AMN.*

Prognosis + histopathology	Additional findings	Additional treatment after 1st surgery	Status
No potential for recurrence i.e., curative (n = 24)			
LAMN (n = 22)	Free margins + intact appendix (n = 24 cases)	none	Alive with no recurrence
Appendiceal adenoma (n = 2)			
Low recurrence risk (n = 8)			
LAMN (Involved margins, Intact mucocele) (6 cases)			
With acellular mucin (2 cases)	Acellular mucin, no neoplastic epithelium (2 cases)	Close follow up (2 cases)	One lost to follow up within 1 year; second followed for 2 years, no recurrence
With neoplastic epithelium (4 cases)	Mucin, neoplastic epithelium (4 cases)	Excision of appendicular stump (developed tumor at stump appendix) (1 case)	Alive, followed for < 1 year with no recurrence, then lost to follow up
		Right hemicolectomy (3 cases)	Followed between 1 and 7 years, all no recurrence
Appendiceal adenoma (2 cases)	Involved margins + intact mucocele (1 case)	Partial cecectomy (dysplasia at resection margin) (1 case)	Followed for < 1 year with no recurrence, then lost to follow up
	NET (carcinoid) + mucinous cystadenoma (1 case)	Right hemicolectomy (1 case)	Followed for 4 years, no recurrence
High recurrence risk (n = 18)			
LAMN (8 cases)	Local perforation or spillage±involved margin	CRS + HIPEC (done) (3 cases)	Patients developed PMP, underwent CRS + HIPEC, followed for 3–6 years after HIPEC, all no recurrence
		CRS + HIPEC (not done^a^) (3 cases)	2 patients followed for 2 years, both had recurrence. 1 patient followed or 4 years, no recurrence.
		Right hemicolectomy^b^ (1 case)	Followed for 1 year, no recurrence
		Close follow up^c^ (1 case)	Followed for 1 year, no recurrence
LAMN + pseudomyxoma peritonei (5 cases)	PMP or peritoneal seeding during surgery or follow up	CRS + HIPEC (done) (2 cases)	Followed for 3–5 years, no recurrence post CRS + HIPEC
		CRS + HIPEC (not done) ^*d*^ (3 cases)	Followed for 0.5–2 years, then lost to follow up
Mucinous adenocarcinoma (5 cases)	PMP or peritoneal seeding during surgery or follow up	CRS + HIPEC (done) (4 cases)	Followed for 2–6 years post CRS + HIPEC, 2 patients had no recurrence, other 2 developed recurrence
		CRS + HIPEC (not done) ^*e*^ (1 case)	Followed for 1 year then lost follow up

^*d,e*^ MDT decision was to undertake CRS + HIPEC for those cases but the procedures were not undertaken because the procedure is not performed in our institution and hence patients were referred abroad with regular follow up.

*Curative, low risk, high risk according to AJCC 8th edition [16], and the PSOGI 2016 classification consensus of mucinous neoplasia of the appendix [4]; LAMN: low appendicular mucinous neoplasm; CRS: cytoreductive surgery; HIPEC: hyperthermic intraperitoneal chemotherapy; PMP: pseudomyxoma peritonei; NET: neuroendocrine tumor.

a MDT decision was to undertake CRS + HIPEC for these 3 cases but the procedures were not undertaken because the procedure is not performed in our institution and hence patients were referred abroad.

b Right hemicolectomy was undertaken as the previous consensus was to perform completion surgery for positive margin.

c Close follow up was undertaken due to recent changes in the guidelines suggesting watchful waiting management as possibility in acellular mucin spillage.

**Fig. 1:**
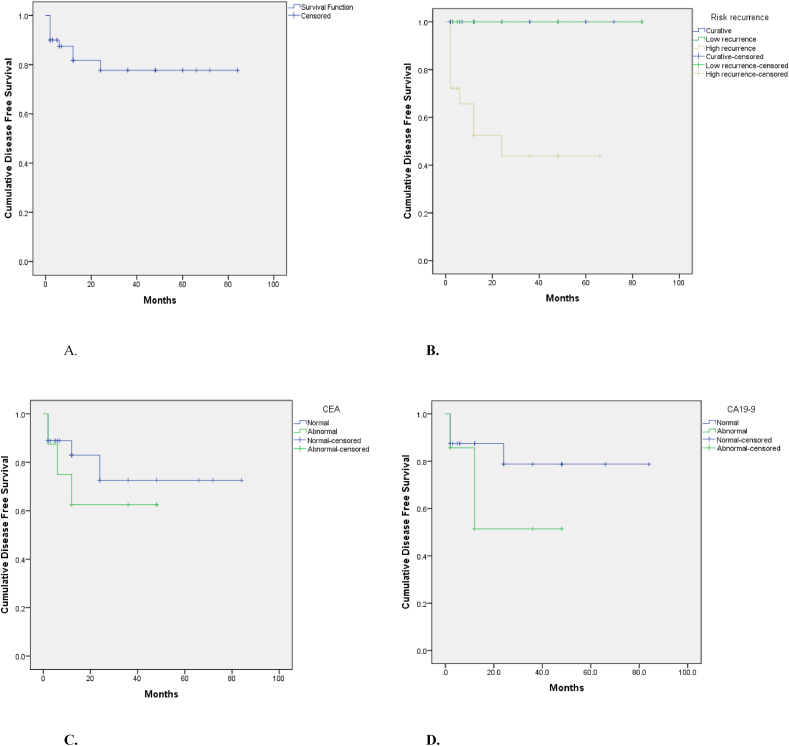
Kaplan–Meier curves showing **A.** Overall disease-free survival (months) for all 50 patients; **B**. Disease-free survival stratified by 3 prognostic categories of recurrence risk: curative (n = 24), low recurrence risk (n = 8), and high recurrence risk (n = 18) (P value < 0.001); **C.** Disease-free survival of patients with normal and abnormal CEA (P = 0.445); and **D.** Disease-free survival of patients with normal and abnormal CA 19-9 (P = 0.117).

Fig. 1 represents the DFS Kaplan–Meier curves. The overall DFS (Fig. 1 A) was about 77% over the whole follow up period (7 years), and none of the patients passed away. When the DFS was computed for each prognostic group, it was significantly lower for the high risk of recurrence group (around 45%) compared to the curative and low risk groups (100%) (P < 0.001) over the 7 years follow up (Fig. 1 B). DFS was lower in patients with higher levels of tumor markers, but the difference was not statistically significant (Fig. 1C and D).

## 5 Discussion

To the best of our knowledge, the current study could be the first from MENA region to discuss the epidemiological profile, imaging aspects, histological and tumor characteristics, and clinical outcomes and prognostic factors of LAMN. The study assessed 12454 patients that underwent appendectomies at our institution in Qatar. We observed 50 patients with AMN (0.4%), in agreement with the literature where AMN represents 0.2–0.7% of all appendix specimens [18].

In terms of demographics, the mean age of the 50 patients in the current study was 47.2 ± 13.1 years, slightly younger than the age range 50–60 years reported in several studies [12]. As for gender distribution, we observed an almost equal gender distribution of AMN with a marginal increase of males over females (1.2:1), in contrast to others that suggested a female predominance (4.1:1) [12]. Our observed higher male prevalence could be attributed to the unique sociodemographic structure of the population in Qatar, where there is a much higher proportion of males over females due to the immigrant worker population which represents 94% of Qatar's workforce and 70% of its total population [19].

As for presentation, many of the patients in the current sample presented with localized right lower quadrant pain (83.3%), localized tenderness (72.5%) and other symptoms suggestive of appendicitis, although nonspecific to AMN. Many of our patients had no fever (6.97%), leukocytosis (48.7%) or palpable mass (17.9%). Our 83.3% abdominal pain is higher than 10–30% abdominal pain reported elsewhere [12,20]. Many of our cases had the diagnoses established either intraoperatively or following post-surgery histology examination, in line with others, where a pre-operative diagnosis was not possible in >50% of the patients [12,20]. Moreover, 4 of our 50 patients (8%) were found to have appendicular mucocele incidentally without any presenting symptoms related to AMN, and another 2 patients presented with symptoms and imaging findings suggestive of ovarian cystic neoplasm, one of the pathologies known to mimic appendicular mass, and were subsequently found to have AMN on post-operative histology examination. These findings agree with evidence suggesting the incidental and atypical presentation of AMN [[20], [21], [22]].

In terms of investigations, CT abdomen was the most used imaging method for diagnosis in the current study (35 out of 44 patients, 79.54%); however, it identified a mucocele in only 54.3% of the patients who underwent CT. These findings agree with recent reports, e.g. a study in Finland that suggested that CT cannot be used to exclude neoplastic etiology underlying acute appendicitis [23]; and similarly, research in Korea found that CT was able to diagnose AMN in only 39 out of 54 suspected patients with positive and negative predictive values of 71.4% and 20% respectively [24].

As for management, there is a lack of a standardized treatment approach for AMN confined to the appendix with no initial apparent metastasis. While some authors recommend the open technique if mucocele is suspected [25,26], studies comparing the best approach suggested that laparoscopic resection without spillage or rupture is feasible and appropriate [26]. At our institution, laparoscopic exploration and resection is the standard technique of treating non metastatic disease (used in 56% of the patients). However, we converted to open technique in 3 (6%) patients due to adherent, mass forming mucocele where conversion to open with adhesiolysis was necessary to resect the mucocele with no spillage or rupture. Right hemicolectomy was done for 4 patients as primary surgery due to cecal involvement or large adherent mass.

Regarding margin involvement after primary surgery, positive margins were observed in 11 patients (23.4%, 6 patients post laparoscopic appendectomy and 5 patients post open appendectomy). For 2 of these 11 patients, the histopathological reports indicated mucinous adenocarcinoma and those patients underwent secondary surgery (CRS + HIPC), particularly that the TNM staging for those patients was T4 M1. A third patient with margin involvement had a rare neuroendocrine tumor (carcinoid) accompanied by mucinous cystadenoma, for whom we undertook completion right hemicolectomy due to margin involvement by the carcinoid tumor and patient was followed for the next 4 years with no signs of recurrence. In this rare carcinoid case, our management is in concord with recent reports suggesting that the management and follow-up of appendiceal combined tumors requires a collective consideration of the involved histological tumor types, especially that the most aggressive component is the one that metastasizes and determines the evolution of the disease [27].

The remaining 8 patients with margin involvement had LAMN. For 6 of these patients, we proceeded with secondary surgery due to spillage or locally advanced disease. The other 2 patients with intact mucocele were managed via close follow up (Table 5); one of them was lost follow up within 1 year and the other was followed for 2 years with no recurrence. For both these patients, our management considered the lack of signs of neoplastic epithelium in the proximal margin, as only acellular mucin was found. Despite that some authors perform completion right hemicolectomy in patients with a positive surgical margins after appendectomy for an intact LAMN [28], we followed the updated guidelines of the American Society of Colon and Rectal Surgeons, as well as others who suggest that involvement of the appendectomy margins by neoplastic epithelium or acellular mucin do not predict recurrence of the disease, hence completion hemicolectomy is not advocated in such patients with a microscopically positive resection margin [10,29].

While appendectomy with free margin is a sufficient treatment for patients with AMN confined to the appendix, there remains no consensus regarding management of patients with local perforation or spillage with or without positive margins [3]. If the spillage deposits consist of acellular mucin only with no epithelial cells, the recurrence rate is estimated to be between 3 and 7% [9]. This low recurrence rate encouraged many authors to support the close follow up approach for selected patients as no additional benefits were accrued from right hemicolectomy over appendectomy alone [3,30].

Out of 8 (16%) patients with appendiceal perforation or local spillage, we had one patient with local spillage of acellular mucin who was closely followed up for 1 year with no recurrence. The other 7 patients were not suitable for follow up alone as they all had spillage of cellular mucin or neoplastic cells and the decision was to proceed with secondary surgery. However, 3 of these 7 patients did not undertake surgery (reasons detailed in Table 5). Two of these 3 patients had recurrence within 2 years; but surprisingly, the third patient diagnosed with perforated mucocele and cellular mucin spillage who did not undertake secondary surgery, did not show recurrence at the 4 year follow up. The MDT decision for this patient was to proceed with CRS + HIPEC, but the patient nevertheless preferred conservative follow up over secondary surgery.

Pseudomyxoma peritonei (PMP) or peritoneal seeding were found during primary surgery or follow up of 10 patients. Five of these 10 patients had a final histopathological diagnosis of LAMN, the other 5 had histopathological diagnosis of mucinous adenocarcinoma. These 10 patients were referred for CRS + HIPEC. However, 2 of the 5 patients with PMP following LAMN who undertook CRS + HIPEC did not have clinical or radiological recurrence until last follow up (3–5 years). In contrast, 2 out of 4 patients diagnosed with PMP following mucinous adenocarcinoma who underwent CRS + HIPEC had recurrence during follow up (50% rate) (Table 5). Such finding of higher recurrence rate for PMP following mucinous adenocarcinoma compared to PMP following LAMN agrees with the previous literature stating that PMP due to carcinoma has higher rates of recurrence and less overall survival than PMP due to peritoneal adenomucinosis [31,32]. None of our 10 PMP patients passed away due the disease progression during the follow up period.

The average length and width of the submitted appendiceal mucocele were 5.9 ± 4.5 cm and 4.25 ± 4.2 cm respectively (Table 4). There was no association between the average size of the mucocele and the prognosis, as only 1 out of the 5 appendiceal mucinous adenocarcinoma (worst prognosis) was larger than the average size of our sample (15 cm length and 15 cm width). Such lack of association between the size and prognosis of the mucocele supports other studies that suggested no significant statistical association between size and prognosis of AMN [33].

For the current sample, the histopathological findings after the primary surgery confirmed LAMN as the dominant type (82%) and mucinous adenocarcinoma was identified in only 10% of the patients, supporting a recent review that proposed that most AMNs are originally derived from LAMN [31]. Hence, LAMN could be considered an adenomatous change in the appendiceal mucosa [31]. Less commonly, AMN may arise from an adenomatous colonic polyp and/or serrated adenoma [31].

In terms of prognosis, the AJCC 8th edition and the PSOGI 2016 classification consensus categorized AMN into three risk of recurrence categories: those that are cured by surgery; AMN with low risk of recurrence; and, AMN with high risk of recurrence [4,16]. Employing the same classification, the distribution of our cases agrees with the AJCC risk of recurrence report (Table 5), as none of our patients who fell in the curative (appendix removed intact, no involved margins) or low recurrence risk (intact appendix, involved margins) groups had recurrence.

In terms of survival, the overall survival across our sample was 100%; no patients passed away due to disease progression during the follow up (7 years for some patients) (Table 4). The Kaplan Meier plot (Fig. 1 A) demonstrates the overall DFS for all the 50 patients, it's less than 100% and this might be attributed to the recurrence in the high recurrence risk group, as none of the patients in the curative or low recurrence risk groups had recurrence after treatment completion (Fig. 1 B). depicts the DFS Kaplan Meier curve based on the 3 recurrence risk categories. The DFS was significantly higher (P < 0.001) for both the curable and low recurrence risk groups compared to the high recurrence risk group, as half the patients in high recurrence risk group exhibited recurrence during follow up either after initial surgery or after CRS + HIPEC. This is in agreement with the AJCC 8th edition and PSOGI 2016 consensus [4,16].

As regards to the association between the DFS and tumor markers (CEA and CA 19-9), patients with elevated ≥ 1 tumor markers had less overall DFS, however the relationship did not reach not statistical significance (P = 0.445 and 0.117 respectively, Fig. 1C and D). This is in partial agreement with previous reports where CEA, CA 19-9 and CA 125 were elevated in patients with recurrence [34]. This suggests that larger sample sizes may be needed in order to detect the association of tumor markers with disease recurrence [34,35].

## 6 Conclusion

AMN is a rare pathology of the appendix. However, cases of atypical appendicular pathology require a high index of suspicion to avoid the risk of a missed AMN progressing to pseudomyxoma peritonei. A laparoscopic approach for AMN may be feasible. The PSOGI prognostic categories were correlated with the recurrence risk and hence may be useful in predicting the prognosis. Unlike previously thought, AMN with acellular mucin at margin or local acellular mucin spillage may not require secondary surgery, particularly among low risk of recurrence patients. Tumor markers may predict the recurrence risk but require large sample sizes. Further multi-center research is required to address the optimal management of positive margins.

## Ethics approval

All the information was retrospectively retrieved from the chart review and patients are de-identified, this study was approved by Medical Research Center, Hamad Medical Corporation reference number (IRB#17167/17).

## Source of funding

Nothing to declare.

## Authors' contributions

A Aleter: data collection, interpretation, writing the paper; W EL Ansari: data interpretation, writing the paper; A Toffaha: data collection, interpretation, writing the paper; A Ammar: pathology data collection, editing the paper; F Shahid: data collection, editing the paper; A abdelaal: study concept, data interpretation, editing the paper.

## Research registration number

Name of the registry: research registry.

Unique Identifying number or registration ID: research registry6571.

Hyperlink to the registration (must be publicly accessible): https://www.researchregistry.com/browse-the-registry#home/registrationdetails/6027e8ff0a2dc1001b76c8f7/

## Guarantor

Prof Dr Walid El Ansari: welansari9@gmail.com.

## Consent for publication

All involved authors consented for the publication of this paper.

## Declaration of competing interest

The authors declare no conflicts of interest.
